# Supplementary material for: Validation of the German Emotional Contagion Scale and development of a mimicry brief version
Source: PLoS One. 2025 Sep 9;20(9):e0331953. doi: 10.1371/journal.pone.0331953 (PMC12419621; doi:10.1371/journal.pone.0331953)
Supplement: S1 File — Study 1 CFA results with the DWLS estimation method. S2. Results for an ECS version including items 06, 09, and 12. S3. Data Study 1. S4. Data Study 2. S5. Data Study 3. S6. ECS items.S7. Power analyses.S8. CCCs study 2. S9. CCCs study 3. (ZIP) [file pone.0331953.s001.zip › Supporting Information/S1.docx]

**S1 Study 1 CFA Results with the DWLS estimation method**

| Scale | Model | χ^2^ | df | CFI | TLI | RMSEA | SRMR |
| --- | --- | --- | --- | --- | --- | --- | --- |
| ECS-Total | 1F | 166.505 | 54 | .747 | .691 | .104 | .110 |
|  | 1F+CE | 73.661 | 42 | .929 | .888 | .062 | .072 |
|  | BF | 36.310 | 36 | .999 | .999 | .007 | .050 |
| ECS-Short | 1F | 4.361 | 2 | .925 | .774 | .078 | .050 |
