# Supplementary material for: Validation of the German Emotional Contagion Scale and development of a mimicry brief version
Source: PLoS One. 2025 Sep 9;20(9):e0331953. doi: 10.1371/journal.pone.0331953 (PMC12419621; doi:10.1371/journal.pone.0331953)
Supplement: S1 File — Study 1 CFA results with the DWLS estimation method. S2. Results for an ECS version including items 06, 09, and 12. S3. Data Study 1. S4. Data Study 2. S5. Data Study 3. S6. ECS items.S7. Power analyses.S8. CCCs study 2. S9. CCCs study 3. (ZIP) [file pone.0331953.s001.zip › Supporting Information/S2.docx]

**S2 Results for an ECS Version Including Items 06, 09, and 12**

All analyses were repeated for an ECS version including the items addressing love (06, 09, and 12) in addition to the other twelve items. In the following, the results for a score summing up all 15 original ECS items (as translated by Falkenberg, 2005) can be found (referred to as *ECS with love*; ECS-WL).

**CFA (All Studies)**

Table 2

*CFA Results of Studies 1-3 (Satorra-Bentler Corrected Test Statistic in Parentheses)*

| Scale | Study | Model | χ^2^ | df | CFI | TLI | RMSEA | SRMR |
| --- | --- | --- | --- | --- | --- | --- | --- | --- |
| ECS-WL | 1 | 1F | 351.203 (326.362) | 90 | .503 (.504) | .420 (.422) | .122 (.116) | .121 (.121) |
|  |  | 1F+CE | 154.589 (144.698) | 75 | .849 (.854) | .788 (.795) | .074 (.069) | .096 (.096) |
|  |  | BF | 81.320 (76.601) | 65 | .969 (.976) | .950 (.961) | .036 (.030) | .049 (.049) |
|  |  |  |  |  |  |  |  |  |
|  | 2 | 1F | 885.753 (805.972) | 90 | .492 (.475) | .407 (.387) | .141 (.134) | .120 (.120) |
|  |  | 1F+CE | 234.607 (218.002) | 75 | .898 (.895) | .857 (.853) | .069 (.066) | .076 (.076) |
|  |  | BF | DNC |  |  |  |  |  |
|  |  |  |  |  |  |  |  |  |
|  | 3 | 1F | 407.352 (380.166) | 90 | .556 (.528) | .482 (.449) | .140 (.134) | .128 (.128) |
|  |  | 1F+CE | 151.978 (142.482) | 75 | .892 (.890) | .849 (.846) | .076 (.071) | .078 (.078) |
|  |  | BF | 89.067 (84.711) | 65 | .966 (.968) | .946 (.948) | .045 (.041) | .052 (.052) |
| *Note*. DNC: Model did not converge. Also the love items were allowed to be correlated among each other in the 1F+CE model and were clustered by a factor in the BF model. | | | | | | | | |

**Study 1**

***Internal Consistency***

The ECS-WL reached an α = .73, ω = .79.

***Convergent and Discriminant Validity***

The correlation between the ECS-WL and the ECS-Short was *r* = .78, *p* < .001.

*Associations between ECS-WL and External Measures*

| Measure - Scale | ECS-WL |
| --- | --- |
| IRI - EC | .50*** |
| IRI - PT | .24*** |
| IRI - FT | .40*** |
| IRI - PD | .31*** |
| BES - AE | .65*** |
| BES - CE | .40*** |
| FAB - HG | .16* |
| NEO - A | .24*** |
| NEO - C | .20** |
| NEO - E | .15* |
| NEO - N | .14* |
| NEO - O | .16* |
| KSE-G - PQ | .07 |
| KSE-G - NQ | .12* |

**Study 2**

***Internal Consistency and Psychometric Properties***

The internal consistency of the ECS-WL was α = .78 (ω = .83). The response probabilities of items 06, 09, and 12 were .71, .74, and .84, respectively. The means (standard deviations) of items 06, 09, and 12 were 2.84 (0.87), 2.94 (0.91), and 3.38 (0.69), respectively.

*Factor Loadings and item-total correlations of the ECS-WL*

| Item | 01 | 02 | 03 | 04 | 05 | 06 | 07 | 08 | 09 | 10 | 11 | 12 | 13 | 14 | 15 |
| --- | --- | --- | --- | --- | --- | --- | --- | --- | --- | --- | --- | --- | --- | --- | --- |
| λ_WL_ | .32 | .15 | .17 | .50 | .54 | .10 | .57 | .49 | .24 | .60 | .16 | .09 | .59 | .42 | .57 |
| *rit_WL_* | .44 | .37 | .46 | .57 | .46 | .38 | .47 | .43 | .52 | .49 | .49 | .40 | .46 | .47 | .34 |
| *Note*. λ_WL_: factor loading within CFA (one-factor, correlated error terms among items addressing each emotion; love items also clustered). *rit_WL_:* part-whole corrected item-total correlation. | | | | | | | | | | | | | | | |

***Convergent and Discriminant Validity***

*Associations between ECS-WL and HEXACO dimensions and
GERT-S*

|  | H | E | X | A | C | O | GERT-S |
| --- | --- | --- | --- | --- | --- | --- | --- |
| ECS-WL | -.05 | .48*** | -.04 | .04 | -.00 | .10* | .07 |
| *Note.* HEXACO: Sample 1 (n = 442); GERT-S: Sample 2 (n = 231). | | | | | | | |

***Longitudinal Invariance***

*Series of Invariance Model Comparisons for the ECS-WL across Measurement Occasions (Satorra-Bentler Corrected Indices in Parentheses)*

| Model | χ^2^ | df | *P* | RMSEA | CFI | Δ CFI |
| --- | --- | --- | --- | --- | --- | --- |
| Configural | 2681.561 (2544.491) | 1629 | < .001 | .069 (.065) | .805 (.798) | - |
| Metric | 2738.793 (2582.916) | 1674 | < .001 | .069 (.064) | .803 (.799) | .002 (-.001) |
| Scalar | 2854.071 (2768.864) | 1719 | < .001 | .070 (.068) | .790 (.768) | .013 (.031) |
| *Rel. Int. 05* | 2834.003 (2737.876) | 1716 | < .001 | .070 (.067) | .793 (.774) | .010 (.025) |
| *Rel. Int. 05, 08* | 2823.422 (2720.992) | 1713 | < .001 | .070 (.066) | .795 (.778) | .008 (.021) |
| *Rel. Int. 05, 08, 03* | 2809.787 (2699.568) | 1710 | < .001 | .069 (.066) | .797 (.782) | .006 (.017) |
| *Rel. Int. 05, 08, 03,  04* | 2802.137 (2686.708) | 1707 | < .001 | .069 (.065) | .797 (.784) | .006 (.015) |
| *Rel. Int. 05, 08, 03,  04, 06* | 2787.541 (2663.597) | 1704 | < .001 | .069 (.065) | .800 (.788) | .003 (.011) |
| *Rel. Int. 05, 08, 03,  04, 06, 12* | 2777.684 (2648.103) | 1701 | < .001 | .069 (.064) | .801 (.791) | .002 (.008) |
| Residual |  |  |  |  |  |  |
| *Rel. Int. 05, 08, 03,  04, 06, 12* | 2865.680 (2707.822) | 1746 | < .001 | .069 (.064) | .793 (.788) | .008 (.003) |
| *Note.* Rel: relaxed; Int.: equality constraint of intercepts. The following number(s) refer(s) to the item(s), in terms of which the respective equality constraint was relaxed. | | | | | | |

***Temporal Stability***

*Temporal Stabilities of the ECS-WL*

|  | ECS-Total | |
| --- | --- | --- |
|  | Manifest | Latent |
| 3-4 Months |  |  |
| t1 t2 | .82 | .94 |
| t2 t3 | .76 | .83 |
| t3 t4 | .79 | .91 |
| 6-8 Months |  |  |
| t1 t3 | .75 | .86 |
| t2 t4 | .81 | .93 |
| 9-12 Months |  |  |
| t1 t4 | .81 | .94 |
| *Note.* All *p*s < .001. | | |

**Study 3**

***Convergent and Discriminant Validity***

*Convergent and Discriminant Associations of the ECS-WL*

|  | ECS-WL |
| --- | --- |
| BES - AE | .67*** |
| BES - CE | .37*** |
| TEQ | .66*** |
| ECQ - CA | .29*** |
| ECQ - CD | .33*** |
| ECQ - AA | .38*** |
| ECQ - AD | .35*** |
| ECQ - AR | .47*** |
| NARQ-S - A | .05 |
| NARQ-S - R | -.20** |
| RMET-S | .09 |
