# Supplementary material for: Validation of the German Emotional Contagion Scale and development of a mimicry brief version
Source: PLoS One. 2025 Sep 9;20(9):e0331953. doi: 10.1371/journal.pone.0331953 (PMC12419621; doi:10.1371/journal.pone.0331953)
Supplement: S1 File — Study 1 CFA results with the DWLS estimation method. S2. Results for an ECS version including items 06, 09, and 12. S3. Data Study 1. S4. Data Study 2. S5. Data Study 3. S6. ECS items.S7. Power analyses.S8. CCCs study 2. S9. CCCs study 3. (ZIP) [file pone.0331953.s001.zip › Supporting Information/S6 - ECS items.docx]

**The Emotional Contagion Scale.**

| 1. If someone I'm talking with begins to cry, I get teary-eyed. |
| --- |
| 2. Being with a happy person picks me up when I'm feeling down. |
| 3. When someone smiles warmly at me, 1 smile back and feel warm inside. |
| 4. I get filled with sorrow when people talk about the death of their loved ones. |
| 5. I clench my jaws and my shoulders get tight when 1 see the angry faces on the news. |
| 6. When 1 look into the eyes of the one I love, my mind is filled with thoughts of romance. |
| 7. It irritates me to be around angry people. |
| 8. Watching the fearful faces of victims on the news makes me try to imagine how they might be feeling. |
| 9. I melt when the one I love holds me close. |
| 10. I tense when overhearing an angry quarrel. |
| 11. Being around happy people fills my mind with happy thoughts. |
| 12. I sense my body responding when the one I love touches me. |
| 13. I notice myself getting tense when I'm around people who are stressed out. |
| 14. I cry at sad movies. |
| 15. Listening to the shrill screams of a terrified child in a dentist's waiting room makes me feel nervous. |
| *Note.* Happiness items = 2, 3, & 11; Love items = 6, 9, & 12; Fear items = 8, 13, & 15; Anger items = 5, 7, & 10; Sadness items = 1, 4, & 14. This material stems from Doherty RW. The emotional contagion scale: A measure of individual differences. Journal of Nonverbal Behavior. 1997;21(2):131-54. Springer. <https://link.springer.com/article/10.1023/A:1024956003661>. Reproduced with permission from SNCSC. Please note that the Love items were excluded from the scale within the present research. Please note that the suggested brief version (ECS-Short) includes the items 1, 3, 5, and 13. |
