# Supplementary material for: Validation of the German Emotional Contagion Scale and development of a mimicry brief version
Source: PLoS One. 2025 Sep 9;20(9):e0331953. doi: 10.1371/journal.pone.0331953 (PMC12419621; doi:10.1371/journal.pone.0331953)
Supplement: S1 File — Study 1 CFA results with the DWLS estimation method. S2. Results for an ECS version including items 06, 09, and 12. S3. Data Study 1. S4. Data Study 2. S5. Data Study 3. S6. ECS items.S7. Power analyses.S8. CCCs study 2. S9. CCCs study 3. (ZIP) [file pone.0331953.s001.zip › Supporting Information/S7 - Power analyses.docx]

**Post hoc power analyses**

Power analyses were conducted with G*Power (1) and the R package SemPower (2)

**Study 1**

Based on an n = 195 and α = .05, a Pearson correlation about *r* = .30, .20, .10 could be detected with a power of 99.7%, 88.4%, and 40.3%, respectively.

For the factor analyses, given an n = 195, df = 42, α = .05, and a cutoff of RMSEA = .06, we achieved a power about 82.3%.

**Study 2**

Based on an n = 442 (sample 1) and α = .05, a Pearson correlation about *r* = .30, .20, .10 could be detected with a power of > 99.9%, 99.6%, and 67.9%, respectively.

Based on an n = 231 (sample 1) and α = .05, a Pearson correlation about *r* = .30, .20, .10 could be detected with a power of 99.9%, 92.6%, and 45.2%, respectively.

For the factor analyses, given an n = 442, df = 42, α = .05, and a cutoff of RMSEA = .06, we achieved a power about 99.9%.

**Study 3**

Based on an n = 180 and α = .05, a Pearson correlation about *r* = .30, .20, .10 could be detected with a power of 99.5%, 86.1%, and 38.1%, respectively.

For the factor analyses, given an n = 180, df = 42, α = .05, and a cutoff of RMSEA = .06, we achieved a power about 77.8%.

1. Faul F, Erdfelder E, Buchner A, Lang A-G. Statistical power analyses using G* Power 3.1: Tests for correlation and regression analyses. Behavior research methods. 2009;41(4):1149-60.

2. Moshagen M, Bader M. semPower: General power analysis for structural equation models. Behavior Research Methods. 2024;56(4):2901-22.
