# Supplementary material for: Validation of the German Emotional Contagion Scale and development of a mimicry brief version
Source: PLoS One. 2025 Sep 9;20(9):e0331953. doi: 10.1371/journal.pone.0331953 (PMC12419621; doi:10.1371/journal.pone.0331953)
Supplement: Attachment — Submitted filename: Review PONE-D-25-13429.pdf [file pone.0331953.s002.pdf]

Review

Journal name: PLOS ONE
Manuscript number: PONE-D-25-13429
Manuscript title: Assessing emotional contagion: Dimensionality, nomological network, longitudinal invariance, and 1-year stability of the German emotional contagion scale and development of a brief version
Date received: May 22, 2025
Date of review: June 23, 2025
Suggestion: Major revision

Comments to the Authors

Thank you for the opportunity to review this manuscript!

The manuscript seems promising and especially the development of the presented brief scale could be interesting for future studies.

However, it is a very complex paper with a very large number of reported findings and potential limitations. Thus, it seems necessary that the writing and reporting is particularly consistent, transparent, and understandable!

Overall, I see a number of problems that could be addressed in a major revision.

Title:
Emotional contagion represents an interpersonal process between two or more individuals. The ECS aims at assessing in individual's susceptiblity to emotional contagion. Hence, "assessing emotional contagion" is misleading and incorrect.

Generally, the name of the ECS scale was misleading from the beginning (it does not measure the process emotional contagion, even if Doherty says so). Further, the reported scale does not contain all items of the ECS. For these reasons, I am not convinced that the reported scale should adapt the initially misleading name ECS. The short version, thus, does not aptly represent a short version of the original ECS, but instead rather seems to represent a "mimicry version" of your modified ECS (German version) which could be an interesting addition for future studies.

Abstract:
To me it seems that a lot of terms are brought up here that are used ambiguously in different areas of research (mimicry, synchronization). For the purpose of clarity, it seems helpful to stick to the central topic of the manuscript (susceptibility to emotional contagion) and prevent the misconception of assessing emotional contagion with the ECS.
It seems unusual to use abbreviations in the abstract.
To avoid misunderstandings, better report sample sizes of the three studies separately.
The findings on the models' fit are misleadingly reported. The fit indices did not support "satisfactory fit" for both the 1F+CE and the BF model.

Introduction:
Again, emotional contagion represents an interpersonal process between two or more individuals. This process should be clearly delineated from an individual's susceptiblity to emotional contagion which seems to be what you aim to investigate in this paper. Please review your use of emotional contagion throughout the manuscript and be more precise about the concept you are investigating.

Further, the conceptual ambiguity of the concept empathy should be discussed and both emotional contagion and susceptibility to emotional contagion should be delineated from empathy and/or different empathy-subfacets/concepts.

You are almost exclusively citing Hatfield and colleagues. Other references should be included on emotional contagion and susceptibility to emotional contagion.

Mimicry has been conceptualized very differently in psychological research regarding socio-emotional processes (eg Hess or Dimberg etc) which should be discussed alongside Hatfield's use of the term.

Explain/justify why you use a model from the context of psychopathy and not a more general model given the non-clinical nature of your samples.

Throughout the introduction, many terms are brought up but not defined, eg empathic concern, emotion awareness. This makes it hard to follow for the reader. Consider briefly defining these concepts.

The selection of discrete emotions in the ECS should be discussed more critically. They do not represent any commonly reported model. Discuss general critique regarding discrete emotion models. Discuss the ambiguity and overlap of some of the items, eg fear with stress.

Other self-report measures of an individual's susceptibility to emotional contagion should be discussed, eg emotion-specific or positively-negatively valenced scales.

The use of a very large number of abbreviations makes it hard to read and follow, eg EC, PT, FT, IRI, etc. Consider dropping the abbreviations and writing the full words instead.

Explain/justify why you still consider your scale(s) a version of the ECS after excluding 3 items only based on your theoretical (but very reasonable) critique on the love items? Can your scale really be called an ECS scale then?
Explain why you kept the rest of the discrete emotions items?

The ECS short rather seems to be a subscale entailing mimicry items and not just a short version. Overall, the idea of a short ECS-mimicry scale is interesting, eg to assess a more salient and observable aspect of the complex process of ec, but this scale could, thus, be more aptly called some sort of mimicry-version of the modified ECS (without the love items), eg susceptibility to mimic emotional expressions.

Study 1:

Explain why the TEQ was not used in study 1.

Alpha = .05 seems to be too high given the number of correlations due to the problem of multiple testing and alpha inflation.

Clarify whether power analyses are in the supplement, if not please add them.

Add example items to the descriptions of the measures (in all studies and for all measures).

Be consistent in using either joy or happiness (eg page 11, line 233).

Why did you use only one subscale of the FAB scale?

For model comparison, the AIC should be used. The analyses, results, and discussion should be revised accordingly.

In general, be more detailed and explicit about the standards for fit indices you are applying. Specify the cutoffs for the fit indices more precisely (with references).

Discuss the model fit parameter of the models 1F and 1F+CE more thoroughly and more critically.

TLI for the BF model is below the cutoff for a good model fit (.95) according to Hu & Bentler (1999), this should be stated and discussed more clearly!

The fit of the ECS short is not acceptable! this should be stated and discussed more clearly.

Alpha and omega for both scales are low, this should be discussed. Provide references for the claim that a response scale comprising 4 answers can/should be considered ordinal.

Correlations should be summarized in the text, "largely in line" is not sufficient here.

The table's format is crooked and out of line.
What is the last column?
In the sample description you say 192 individuals filled out the survey, here you report up to 301 for study 1? Which number is correct?
The title of the table says study 1 and 3, but the column "study" says 1 and 2.

Were the measures normally distributed?
Please report more detailed descriptive statistics for all measures in all studies.

I am having a hard time with the not-preregistered "predictions". Explain and justify why the analysis of study q (and study 2) apparently were not preregistered?
How do you tackle the uncertainty that arises from the fact that these analyses were not preregistered?

This needs to be discussed.

Study 2:
Explain more precisely why nursing staff was chosen, eg also very high share of women?

Explain/justify why data from 2016 is used.
Was the 2016 study also approved by the ethics committee?

Clarify the intervention conducted in the overarching project.

Add sample information for sample 2 and 3 (eg gender, age, etc) and consider using a table for all three samples.

Clarify (for all studies and all measures), whether only the answers 0 and 4 (or 5) were presented to the participants with words or all answers (i.e., 0, 1, 2, 3, 4, 5). Please provide all answer options that were presented.

Again, for model comparison, the AIC should be used. The analyses, results, and discussion should be revised accordingly.

Again, TLI is below the cutoff for a good model fit (.95) according to Hu & Bentler (1999), this should be stated and discussed more clearly!

Discuss the TLI value > 1 for the ECS short in study 2.

Was ordinal alpha and omega calculated here?
Explain why you consider 4 response options ordinal and 5 continuous (enough)?

Summarize the descriptive statistics and psychometric properties in the text and report them in a table instead of the supplement.

Discuss the model fit of the configural invariance model more thoroughly (RMSEA and CFI are not good) and also report SRMR and TLI.

Justify your choice of delta CFI as index for invariance (why not delta chi square or delta RMSEA)?

Be consistent in your use of the terms, eg residual or strict invariance, in the text and tables.
Be consistent in how you report "rel. int. 05, ...", either in parenthesis or after a comma.

Justify why it seems more acceptable to you to modify the original response scale (4 options) than to keep it, given that internal consistency only "increased slightly".

Study 3:
Does the ethics approval only pertain to study 3? Were study 1 and 2 also approved by the ethics committee? Please clarify.

Explain why the preregistration was registered on april 8 and the data collection started in april 4 2024, but it says "preregistered before data collection" in the text.

Discuss the large share of women in the sample (in all three studies). Consider conducting sensitivity analyses for all studies regarding participants' gender.

Clarify whether all response options were presented to the participants with words or only the extremes (for all measures).

Add example items for all measures in all studies.

Again TLI below .95 is not excellent.

Report the AIC and compare the models accordingly (for all studies and model comparisons).

In the preregistration it says that one item was reformulated and tested. I cant find anything on that analysis step in the manuscript. Please clarify.

The potential influence of low alpha values (eg as low as .19) on the correlations should be discussed.
Better report and discuss confidence intervals for all correlations in all studies.

Overall, consider summarizing results and discussion in a combined "Results & Discussion" section (for all studies).

General Discussion:
The dimensionality section of the discussion needs to be revised:
The model fit of the 1F+CE model was not "close to the threshold for a good fit in studies 1 and 3" and the TLI was not excellent either in study 2.
The model fit of the BF model was only excellent in study 2, but not in study 1 and 3 (TLI < .95).
The findings should be discussed accordingly.

The model comparisons should be based on the AIC. The results should be reported transparently and discussed accordingly.

In the convergent and discriminant validity section of the discussion, a concluding remark is missing regarding your evaluation of the scales' validity.

The longitudinal analyses section of the discussion might need to be revised depending on the revised findings, especially regarding the missing model fit indices.

Emotional contagion is not a personality trait (line 631). It is an interpersonal process. What you are investigating is an individual's susceptibility to emotional contagion.

In line 632, "Hatfield et al." seems to be wrong.

The limitations section of the discussion is very brief:
Discuss the risk of alpha inflation due to multiple testing for all studies more thoroughly.
Discuss the samples' homogeneity in all three studies more thoroughly (women, profession, etc).
Discuss the different time points of the three studies with respect to the COVID-19 pandemic more thoroughly (before, during, and after the pandemic) and discuss the potential impact of the time point of study 2 (2016) being 6/8 years before the time points of studies 1 and 3.
Discuss the lack of preregistrated analyses in study 1 and 2.

Include practical implications of your research in the discussion.

Language and Style:
Some commas are missing.
Sometimes blank spaces are missing between parentheses.
APA level 2 headings are written in title case.
Paragraphs should be indented consistently.
Sentences should not start with numbers.
