# Supplementary material for: Validation of the German Emotional Contagion Scale and development of a mimicry brief version
Source: PLoS One. 2025 Sep 9;20(9):e0331953. doi: 10.1371/journal.pone.0331953 (PMC12419621; doi:10.1371/journal.pone.0331953)
Supplement: Attachment — Submitted filename: Response to Reviewers.docx [file pone.0331953.s004.docx]

Dear Dr. Larionow,

Thank you very much for taking our manuscript into consideration and the invitation to resubmit a revision. We would especially like to thank you and the reviewers for the valuable feedback and comments. We believe the revised manuscript to be substantially improved. We explicitly addressed each point in the table below.

Yours sincerely,

The Authors

|  | **Editor‘s / Reviewer’s suggestions** | **Response** |
| --- | --- | --- |
|  | **Journal requirements** |  |
| 01 | Please ensure that your manuscript meets PLOS ONE's style requirements, including those for file naming. | We ensured to meet PLOS ONE's style requirements and also checked the file names. |
| 02 | We note that there is identifying data in the Supporting Information file < Supporting Information.zip>. Due to the inclusion of these potentially identifying data, we have removed this file from your file inventory. Prior to sharing human research participant data, authors should consult with an ethics committee to ensure data are shared in accordance with participant consent and all applicable local laws. | Thank you for highlighting this point. We have now removed all demographic variables from the data frames (S4, S5, S6) and uploaded the <supporting information.zip> again, which should thus now be fully anonymized. |
|  | **Reviewer #1:** |  |
|  | I would like to thank you for inviting me to review this manuscript. I have read the article carefully. While it covers an interesting topic, some concerns lead me to recommend major revisions. My reasons for this decision are as follows: | Thank you very much for taking the time to review our manuscript. |
| 03 | The title mentions the nomological network, but the abstract does not explicitly address it. Including a brief explanation of how the ECS fits within the broader theoretical framework or its relationships with other constructs would enhance the depth of analysis. | We understand that we used the term “nomological network” a bit too careless. Indeed, we wanted to refer to the concept of convergent validity, which is examined in terms of correlations to external measures. We have revised the wording in the title and the whole manuscript to avoid any misunderstandings associated with using the term “nomological network”. |
| 04 | While factor models are mentioned, a brief explanation of the dimensionality and what specific dimensions are being assessed could provide clearer insights into the scale's structure. | We added a sentence in the abstract, clarifying that the mentioned fit of the factor models suggests factorial validity, since the ECS is aimed to measure a general factor. |
| 05 | Adding a sentence about the practical implications or applications of the ECS-Total and ECS-Short could help readers understand the significance of the research beyond its theoretical contributions. | Thank you for highlighting this point. We added a sentence in the abstract, emphasizing the theoretical implications and practical applications of the present research. |
| 06 | More details on the selection process and rationale for the items included in the ECS-Short could be beneficial, especially considering its development is a key aspect of the study. | For the ECS-Short we selected the four items of the total questionnaire that explicitly address the process of mimicry. We revised the wording in the abstract to point this out more clearly. |
| 07 | While the introduction provides a solid foundation, it could benefit from a deeper exploration of prior studies concerning the ECS's psychometric properties in different cultural contexts to strengthen the rationale for the current study. | Thank you for this suggestion. We added details about prior validation studies’ findings on the dimensionality and convergent validity of the ECS in order to provide the reader with a clearer and more comprehensive background of the present research. |
| 08 | The introduction could clarify the distinction between ECt and other components of empathy more explicitly, potentially by providing more examples or discussing the implications of these distinctions in greater detail. | Thanks for this suggestion as well. We revised the subsection that delineates empathy and emotional contagion, now providing deeper insights into the conceptual differences and implications. |
| 09 | The introduction briefly mentions the nomological network but does not delve into what this entails specifically in the context of emotional contagion. Expanding on this concept could provide a clearer understanding of its relevance and importance. | As stated above, we understand that we may have been using the term “nomological network” too loosely. We thus revised our wording in the manuscript as well as the title to refer to the concept of convergent and discriminant validity, which is examined via the correlations to external measures. |
| 10 | Including references to relevant interdisciplinary research (e.g., from neuroscience or sociology) could deepen the analysis and demonstrate the broader implications of studying emotional contagion. | We have added references to relevant interdisciplinary research, e.g., neuroscience, biological / physiological psychology, political psychology to provide the reader with a more comprehensive picture of the broad relevance of emotional contagion. |
| 11 | While the discussion provides a good analysis of the ECS, it could benefit from a more detailed comparison with other emotional contagion measures or related scales to highlight its unique contributions or advantages. | Thank you for your suggestion. We added comparisons to other self-report measures of SEC including references in the general discussion. Please note that there are not many other such questionnaires available and except one, they merely address our construct of interest with one subscale among others, but we anyway connected our findings to the findings reported for these questionnaires. |
| 12 | The discussion could be enhanced by elaborating on the broader implications of the findings for the field of psychology, particularly in understanding emotional contagion and its measurement. | We complemented the general discussion by a last section, pointing out to the implications of the present findings for the field of psychology, as well as the practical applications of the scales examined. |
| 13 | Including potential practical applications of the ECS and ECS-Short in various settings (e.g., clinical, organizational) would extend the discussion's relevance. | We added potential practical applications at the end of the general discussion. |
| 14 | While the analysis of dimensionality is thorough, further clarification on the theoretical justification for choosing the bifactor model over others could strengthen the argument. | Thank you for this suggestion. We added a more detailed explanation for the theoretical justification of the Bi-Factor model over other models in the dimensionality section of the general discussion. |
| 15 | The discussion could integrate more recent studies or theories to support or contrast the findings, providing a more comprehensive literature backdrop. | We complemented the discussion by a more comprehensive comparison with relevant previous studies. |
|  | **Reviewer #2** |  |
|  | Overall. Thank you for this very fascinating series of emotional contagion scale (ECS) studies, which may offer significant contributions both theoretically (factor structure and short form) and practically (a German adaptation). Most notably, the bi-factor model fit and mimicry item short form are theoretically compelling. Also, confirmation of emotional contagion as a 'trait' adds to the theoretical database. Practically, having documented a German language adaptation may be of practical value to clinical and research practitioners. Below I offer some thoughts about your research and manuscript, which you may consider for revision. | Thank you very much for your time and the appreciation you expressed. |
| 16 | The full title is excessively descriptive and yet inadequately describes your research. Your research is very extensive, so describing it in a title seems challenging. Given the theoretical value of your studies, not to mention the German adaptation, I recommend using the short title emphasizing 'validation', a 'German' version, and 'a brief form'. Also, the full title refers to 'nomological network', which is I think may get lost in the manuscript as you do not identify any of your studies as such. | Thank you for highlighting this point. We have shortened the title, while trying to keep the most important information. We also omitted the term “nomological network”, which understandably may have been misleading at this point. |
| 17 | The abstract provides research details (theoretical purpose, design, findings), but presumes some valuable implication. Though limited with respect to words, a comment about theoretical and practical value may enhance the abstract, even if it requires abbreviating other parts. | Thank you for your suggestion. We added a sentence in the abstract, emphasizing the theoretical implications and practical applications of the present research. |
| 18 | Overall. The theoretical framework is clearly provided. Essential constructs and variables are identified along with the purpose(s) of the current research. 1. Comment. When introducing the current research, the section 'The Present Studies' states the purpose is ". . . aimed at validating the German version of the ECS, including its factor structure, nomological network, longitudinal measurement invariance, and temporal stability." Developing and validating a brief version is described as a second purpose. In your introductory paragraph you reference and describe 'three studies'. You then proceed with presentation of each study 1, 2, and 3, each complete with statement of purpose, Method, Measures, Results, and Discussion. 2. Suggestion. The presentation of these studies as described above is confusing. There are 3 studies, each essentially valuable theoretically, yet the actual purpose of each is not clear until the reader has completed reading the entire series of studies. I suggest reconsider presentation of the studies and corresponding results. (a). Prior to presenting the actual studies, in the general introduction to the studies (The Present Studies), make clear briefly what each of the three study purposes are. Maybe have each study be subheaded with the purpose and design/methods. Then, have a Results section, with subheaded studies, for each a brief restatement of purpose followed by the results. Then, have a Discussion section, again, each study subheaded with corresponding Discussion. (b). One confusion pertains to your summary tables, e.g., Table 1, Table 2, each of which have the results for the different Studies, though they are presented within Study 1. My suggestion is basically a reorganization of studies such that the studies 1, 2,3 are nested with (a) purposes and methods, (b) Results, and (c) Discussion. | Thank you for the appreciation you expressed and for your suggestions. We added a specific description of the three studies’ purposes in the general introduction (subsection “The present studies”). We first tried to implement your suggestion to reorganize the presentation of the three studies as nested within (a) purposes and methods, (b) Results, and (c) Discussion. However, this confronted us with another issue: In our present case, the studies were not conducted parallel to each other, but consecutively. Therefore, the purpose and hypotheses of each study build upon the results and discussion of the previous study. Nesting the studies in the section as you described would eliminate this logical connection between the studies. In our eyes, this would bear the risk of reduced readability for the reader, since the purposes and hypotheses of study 2 (3) directly refer to the results and conclusions of study 1 (2, respectively). Nevertheless, this suggestion would in our eyes be of great applicability if the studies would pursue mutually exclusive goals (e.g., only one study focusing on the dimensionality, one on the convergent validity, stability, etc.) that are not consecutively logically linked among each other. Apart from that, thanks also for noting that the tables are not perfectly presented. We agree and now present the tables separately for each studies’ results in order to increase readability. |
| 19 | Overall. Your methods are generally complete with ample description of the models and modeling procedures. 1. Recommendation. While you may be applying generally accepted procedures used in cited research, consider offering a citation of standards for language adaptation of clinical measures and corresponding research designs, e.g., International Test Commission (ITC) and standards for measurement language adaptation. https://www.intestcom.org/. | Thank you for this suggestion. We reviewed the ITC guidelines and can assure that the translation procedure was performed in accordance with these guidelines. We added a sentence clarifying this matter and citing the guidelines. |
| 20 | 2. Recommendation. Study 1 forms used a 4-point Likert response scale, Study 2 forms expanded the response scale to a 5-point Likert. This is a nontrivial change to the forms, one which may go beyond simply obtaining higher internal consistency. At least, some discussion may be needed to explain the process underlying this finding. Furthermore, changing the response format may in fact change the measured construct. Fitting an item response model to the data to understand the response process would be very valuable, particularly for the brief form of mimic items. Note, I am not necessarily suggesting this for this set of studies as a revision, but without question, more attention is called for. | Thank you for emphasizing this point. We agree that some more discussion regarding the response scale is absolutely appropriate and included it as well in the discussion section of study 1 as in the introduction and discussion sections of study 2. The internal consistency did indeed not vary particularly strongly between both studies and response scales. However, as we now explain, a five-point scale is, in our eyes, from a theoretical perspective preferable, as it avoids forcing participants to convert an actual neutral response into a high or low response, potentially increasing error variance and thereby undermining reliability and validity. We also included a reference to a recent meta-analysis, which points out the superiority of response scales using an odd number of categories (and thereby including a middle option, such as the five-point scale does) over response scales using an even number of categories. We agree that IRT analyses would be very interesting, but we are also aware of the complexity of such analyses. Therefore, we decided not to conduct them in the present study, since we deemed a thorough IRT investigation beyond the scope of the present paper, which is already long, to some extent complex and entails many analyses and findings. However, we point out to IRT analyses as a potential future avenue at the end of the general discussion. |
| 21 | 3. Comment. For the convergent and discriminant validity studies, beyond a visual inspection of the measurement correlation matrices with respect to the hypothesized covariances, a more rigorous structural equation model should be fit for hypothesis testing. On the other hand, given the obtained data, fortunately for the researchers, the 'interocular traumatic test' suffices, i.e., you know it when it hits you between the eyes. Again, however, an SEM model could have been fitted to the matrices to test the hypotheses. One of countless relevant citations is, Raykov, Tenko (2011). Evaluation of convergent and discriminant validity with multitrait-multimethod correlations. British Journal of Mathematical and Statistical Psychology, 64, 38-52. | Thank you for this suggestion. We have considered reporting SEM analyses, but detected some problems, while trying to implement your suggestion: The fit of a SEM that includes both the ECS and the other measures is rather poor, likely due to the lack of fit of some of the external measures’ measurement models. For example, instruments like the Interpersonal Reactivity Index or the Reading the Mind in the Eyes Test are well-known to produce inconsistent factor structures. This complicates the SEM, which presupposes a good fit of the single measurement models. As we realized while trying to implement your suggestion, in order to reach a good SEM fit, we would have to implement numerous post hoc modifications first to reach a good fit of the measurement models of the external scales. However, using a large number of post hoc modifications undermines the interpretability of results, especially in light of the fact that the factorial validity of these external scales is not the focus of the present manuscript. We therefore decided not to report SEM and hope that our reasons are understandable. Apart from that, since the focus of the manuscript is to determine the convergent validity of the ECS scale – which is typically scored by summing up item responses instead of running factor analyses – manifest correlations are, in our point of view, an appropriate analysis method. In contrast, a research question concerning the nomological network of emotional contagion as a construct in relation to other constructs would of course be appropriately addressed by an SEM instead of manifest correlations. However, as explained above, we now omit the use of the term *nomological network* consistently throughout our manuscript.  We also added a limitation at the end of the discussion, explicitly pointing out that manifest correlations were computed and SEM would be an interesting future avenue. |
| 22 | 4. Recommendation. Along with validity results, I recommend always providing point reliability estimates with associated standard errors. These would be estimated as parameters using structural equation modeling procedures. | We computed the standard errors for alpha and omega and now report them in addition to the point reliability estimates. |
| 23 | Results. Overall. Results from analyses per study are well described. As noted above, the presentation is sometimes confusing and required several readings to fully appreciate the purpose and results of the series of studies. The abbreviated 'brief' form is most interesting and from some perspective may constitute the most fascinating and certainly original contribution. 1. Suggestion. Table the results for each study independently as described above. | Thank you very much for the appreciation you expressed. We now table the results for each study independently to increase the readability of the results sections. |
| 24 | Overall. There are many findings to be discussed. You return to your research purpose with interpretation of your findings. 1. Comment. The long title mentions nomological network, and while the validity studies may be intended to complete this inquiry, the findings are not discussed as such, i.e., nomological network. This would constitute an important theoretical contribution and findings should be conceptualized in this term. | Thank you again for this note. We understand that we have used the term “nomological network” too careless. As already mentioned, we thus revised our wording in the manuscript as well as in the title. We now refer to convergent and discriminant validity instead, which is analyzed via correlations to external measures. |
| 25 | 2. Recommendation. Highlight the brief form as an original contribution. | We now highlighted the brief form as an original contribution both at the beginning and at the end of the general discussion. |
| 26 | 3. Comment. The implications of psychometric research are sometimes treated as 'needless-to-say'. In this case, you have much to say about implications and should include them. | Thank you for your suggestion. We now explain the implications of the present research at the end of the general discussion. |
|  | Writing. Overall. This is a well written manuscript, and was a pleasure to read. Thank you. | Thank you again very much for your time and your worthwhile comments. We are happy that you liked our manuscript. |
|  | **Reviewer #3:** |  |
|  | Thank you for the opportunity to review this manuscript!  The manuscript seems promising and especially the development of the presented brief scale could be interesting for future studies.  However, it is a very complex paper with a very large number of reported findings and potential limitations. Thus, it seems necessary that the writing and reporting is particularly consistent, transparent, and understandable!  Overall, I see a number of problems that could be addressed in a major revision. | Thank you very much for taking the time to read and review our manuscript. |
| 27 | Emotional contagion represents an interpersonal process between two or more individuals. The ECS aims at assessing in individual's susceptiblity to emotional contagion. Hence, "assessing emotional contagion" is misleading and incorrect. | Thank you for highlighting this point. We basically agree with this view (see below) and deleted this phrase from the title (thereby also shortening the title, as suggested by another reviewer). Moreover, we altered our wording and now refer to susceptibility to emotional contagion as our core construct. |
| 28 | Generally, the name of the ECS scale was misleading from the beginning (it does not measure the process emotional contagion, even if Doherty says so). Further, the reported scale does not contain all items of the ECS. For these reasons, I am not convinced that the reported scale should adapt the initially misleading name ECS. The short version, thus, does not aptly represent a short version of the original ECS, but instead rather seems to represent a "mimicry version" of your modified ECS (German version) which could be an interesting addition for future studies. | As said, we basically agree with your argumentation and renamed our construct of interest from emotional contagion (ECt) to susceptibility to emotional contagion (SEC). We first hesitated to alter the name of the whole questionnaire, questioning whether our contribution would justify such a step. However, after a thorough discussion among the authors we decided to do so in order to be consistent throughout the manuscript. The new name of our adapted questionnaire (where the three love items are excluded) is *susceptibility to Emotional Contagion Scale (sECS)*. However, to avoid confusion, we still refer to Doherty’s original 15-item questionnaire using its original name.  Apart from that, we also renamed the previously called *short version* into *sECS-mimicry*. |
| 29 | Abstract: To me it seems that a lot of terms are brought up here that are used ambiguously in different areas of research (mimicry, synchronization). For the purpose of clarity, it seems helpful to stick to the central topic of the manuscript (susceptibility to emotional contagion) and prevent the misconception of assessing emotional contagion with the ECS. | We revised our wording and now refer to susceptibility to emotional contagion as our core construct. We omitted the definition of this core construct that referred to mimicry and synchronization from the abstract, which was taken from the work of Hatfield and Doherty. |
| 30 | It seems unusual to use abbreviations in the abstract. | We omitted the abbreviations from the abstract. |
| 31 | To avoid misunderstandings, better report sample sizes of the three studies separately. | We now report the sample sizes separately. |
| 32 | The findings on the models' fit are misleadingly reported. The fit indices did not support "satisfactory fit" for both the 1F+CE and the BF model. | Thank you for highlighting this point. We revised the sentence in the abstract to avoid possible misunderstandings. We now point out that we found satisfactory fit of the bi-factor model in all three studies and satisfactory fit of the 1F+CE model in study 2. |
| 33 | Again, emotional contagion represents an interpersonal process between two or more individuals. This process should be clearly delineated from an individual's susceptiblity to emotional contagion which seems to be what you aim to investigate in this paper. Please review your use of emotional contagion throughout the manuscript and be more precise about the concept you are investigating. | We have revised our use of the term *emotional contagion* in the whole manuscript and now refer to susceptibility to emotional contagion (SEC) as our core construct that is aimed to measured by our questionnaire. We also revised the introduction in order to at first provide the reader with information about emotional contagion as an interpersonal process and then later introduce into SEC. |
| 34 | Further, the conceptual ambiguity of the concept empathy should be discussed and both emotional contagion and susceptibility to emotional contagion should be delineated from empathy and/or different empathy-subfacets/concepts. | We added a discussion of the conceptual ambiguity as well the operational heterogeneity of the empathy construct. Furthermore, we now more explicitly delineate emotional contagion from empathy and its subconcepts. |
| 35 | You are almost exclusively citing Hatfield and colleagues. Other references should be included on emotional contagion and susceptibility to emotional contagion. | Thank you for highlighting this point as well. We have added references to other theories and empirical studies to enrich the background of the present study. |
| 36 | Mimicry has been conceptualized very differently in psychological research regarding socio-emotional processes (eg Hess or Dimberg etc) which should be discussed alongside Hatfield's use of the term. | We have now also included a discussion of and references to this social context view on mimicry, which we deem a highly interesting and relevant perspective on mimicry besides Hatfield and colleagues’ view. |
| 37 | Explain/justify why you use a model from the context of psychopathy and not a more general model given the non-clinical nature of your samples. | We agree that alluding to such a model might irritate some reader and excluded it from the manuscript. We instead added a more explicit differentiation between emotional contagion and other empathy subprocesses, such as affective-empathic emotion sharing. |
| 38 | Throughout the introduction, many terms are brought up but not defined, eg empathic concern, emotion awareness. This makes it hard to follow for the reader. Consider briefly defining these concepts. | We added definitions of all concepts, such as empathic concern, to avoid any confusion. |
| 39 | The selection of discrete emotions in the ECS should be discussed more critically. They do not represent any commonly reported model. Discuss general critique regarding discrete emotion models. Discuss the ambiguity and overlap of some of the items, eg fear with stress. | We added several sentences in the introduction (section: content examination of ECS items) that critically reflect the selection of discrete emotion items of the ECS in particular and discrete emotion models in general. We now also point out the conceptual overlap of some items. |
| 40 | Other self-report measures of an individual's susceptibility to emotional contagion should be discussed, eg emotion-specific or positively-negatively valenced scales. | Thank you for this suggestion. We now point out in the introduction that there are also other self-report measures of SEC available. While we conducted the study, we were not aware of e.g. positively-negatively valenced scales, but we think they are a very interesting approach and thus included a discussion of this approach in the general discussion. We think that this discussion is best placed in the general discussion, since we did not include such a measure in our study and therefore cannot explore the correlations / convergent validity between both approaches (which though would in our eyes have been a very enriching addition). We now also refer to other questionnaires of SEC (i.e., measures that include a subscale measuring SEC) in the general discussion and compare our results with the findings reported for these questionnaires. |
| 41 | The use of a very large number of abbreviations makes it hard to read and follow, eg EC, PT, FT, IRI, etc. Consider dropping the abbreviations and writing the full words instead. | We omitted these abbreviations and now use the full words instead. |
| 42 | Explain/justify why you still consider your scale(s) a version of the ECS after excluding 3 items only based on your theoretical (but very reasonable) critique on the love items? Can your scale really be called an ECS scale then? Explain why you kept the rest of the discrete emotions items? | Thank you for stressing this point. As explained above, we renamed our modified scale, from which these three items were excluded, into “susceptibility to Emotional Contagion Scale” (sECS) to avoid possible confusion.  We kept the rest of the discrete emotions items, since they refer to basic emotions as included in established models (e.g., Ekman’s) – which does not apply for *love*. Moreover, those items basically tap the individual susceptibility for the process of “getting infected” with others’ emotions (e.g., “If someone I'm talking with begins to cry, I get teary-eyed.“; item 01). Thus, the raised issues associated with the love items are not in the same way applicable for the other emotion items. Nevertheless, as explained three points earlier (#39), we added a critical discussion of some items‘ content, e.g., the overlap between fear and stress. |
| 43 | The ECS short rather seems to be a subscale entailing mimicry items and not just a short version. Overall, the idea of a short ECS-mimicry scale is interesting, eg to assess a more salient and observable aspect of the complex process of ec, but this scale could, thus, be more aptly called some sort of mimicry-version of the modified ECS (without the love items), eg susceptibility to mimic emotional expressions. | We basically agree with this argumentation and thus renamed the short scale consistently in the manuscript from *ECS-Short* into *sECS-mimicry*. |
| 44 | Explain why the TEQ was not used in study 1. | Study 1 reanalyzed data that had already been collected. Indeed, the TEQ was included in this data set. However, for the aim of the validation of the TEQ, the correlation between the ECS and the TEQ had previously already been published (the article was cited in the manuscript, see methods section of study 1). To ensure that we provide original research only, we therefore omitted this correlation from our present results. |
| 45 | Alpha = .05 seems to be too high given the number of correlations due to the problem of multiple testing and alpha inflation. | We have now adjusted our significance level according to the false discovery rate (FDR). The *p* values and adjusted alphas can be found in the tables that contain the correlational results (which we separated between the studies and sECS versions to increase readability). The pattern of results did not change substantially. |
| 46 | Clarify whether power analyses are in the supplement, if not please add them. | We added power analyses as a new supporting information file (S7). |
| 47 | Add example items to the descriptions of the measures (in all studies and for all measures). | We added example items to the descriptions of all measures from all studies. |
| 48 | Be consistent in using either joy or happiness (eg page 11, line 233). | We now consistently refer to happiness instead of joy. |
| 49 | Why did you use only one subscale of the FAB scale? | We were looking for a brief measure that captures the core concept of altruism as giving help to someone in need. This concept is represented by the *help giving* subscale of the FAB. The other subscales *Moral Courage*  (MC), and Altruistic *Peer Punishment* (PP) were considered less relevant from a theoretical perspective. |
| 50 | For model comparison, the AIC should be used. The analyses, results, and discussion should be revised accordingly. | Thank you for highlighting this matter. We agree and computed AIC values for all models and now consistently report and discuss them in our manuscript. |
| 51 | In general, be more detailed and explicit about the standards for fit indices you are applying. Specify the cutoffs for the fit indices more precisely (with references). | We added a more detailed description of the considered cutoffs for fit indices including references in the CFA results section of study 1. |
| 52 | Discuss the model fit parameter of the models 1F and 1F+CE more thoroughly and more critically. | We revised our whole CFA interpretations in order to discuss the model fit of this model as well as the other models from a more thorough and critical perspective. |
| 53 | TLI for the BF model is below the cutoff for a good model fit (.95) according to Hu & Bentler (1999), this should be stated and discussed more clearly! | We included a clear statement, clarifying that TLI was below the good model fit as suggested by Hu and Bentler in this case. |
| 54 | The fit of the ECS short is not acceptable! this should be stated and discussed more clearly. | We revised our interpretation of the CFA results concerning this scale as well. |
| 55 | Alpha and omega for both scales are low, this should be discussed. Provide references for the claim that a response scale comprising 4 answers can/should be considered ordinal. | We now discuss the consistency values from a more critical perspective. Apart from that, we agree that it may be arbitrary to treat a 4 (but not 5) point likert scale ordinal. We thus now report ordinal alpha and omega for studies 2 and 3 (where a 5 point scale was used) as well. |
| 56 | Correlations should be summarized in the text, "largely in line" is not sufficient here. | We now summarize the correlational results (as fully displayed in the tables) in the text. |
| 57 | The table's format is crooked and out of line. What is the last column? | We revised our tables and paid careful attention to avoid such problems. |
| 58 | In the sample description you say 192 individuals filled out the survey, here you report up to 301 for study 1? Which number is correct? | Study 1 contained 195 participants. We now report this number consistently throughout the manuscript. |
| 59 | The title of the table says study 1 and 3, but the column "study" says 1 and 2. | We revised our tables and paid careful attention to avoid such problems. |
| 60 | Were the measures normally distributed? Please report more detailed descriptive statistics for all measures in all studies. | No, the measures were not normally distributed. We now clarify this explicitly in the main text in the results section of study 1. Moreover, we now also point out to this matter in the note of every CFA and correlations table (also of studies 2 and 3). We have already reported Satorra-Bentler corrected CFA statistics in order to account for a violation of the normality assumption. |
| 61 | I am having a hard time with the not-preregistered "predictions". Explain and justify why the analysis of study q (and study 2 ) apparently were not preregistered? How do you tackle the uncertainty that arises from the fact that these analyses were not preregistered? This needs to be discussed. | Studies 1 and 2 reanalyzed data that had been collected earlier in previous projects (references are cited in the method sections). We did not preregister these two studies, but we preregistered study 3. We added the fact that merely study 3 was preregistered in the limitations section at the end of the discussion. |
| 62 | Explain more precisely why nursing staff was chosen, eg also very high share of women? | We added an explanation in study 2, which points out that nursing as a profession especially depends on empathy and related processes as well as the individual susceptibility of employees for these processes. Therefore, nursing staff is an especially interesting group for studying as well interpersonal empathic processes as the individual susceptibility to such proceses (e.g., emotional contagion).  However, since nursing is a profession with predominantly female employees, it was not possible to simultaneously address the problem of gender imbalance. |
| 63 | Explain/justify why data from 2016 is used. Was the 2016 study also approved by the ethics committee? | We used data from 2016, because it was collected within a larger longitudinal project. Due to the high practical effort to collect data from four measurement occasions each three to four moths apart (i.e., over a whole year), we were not able to repeat the design for this paper, but instead accessed data that were already collected in 2016.  Yes, this study was also approved by an ethics committee, we added a sentence clarifying this. |
| 64 | Clarify the intervention conducted in the overarching project. | We added an explanation in the methods section of study 2. |
| 65 | Add sample information for sample 2 and 3 (eg gender, age, etc) and consider using a table for all three samples. | We created a table as suggested, providing this information. |
| 66 | Clarify (for all studies and all measures), whether only the answers 0 and 4 (or 5) were presented to the participants with words or all answers (i.e., 0, 1, 2, 3, 4, 5). Please provide all answer options that were presented. | We have added a more detailed description of the response scales, now providing all options that were presented. |
| 67 | Again, for model comparison, the AIC should be used. The analyses, results, and discussion should be revised accordingly. | We agree and now report and discuss AIC values consistently through all CFA in the manuscript. |
| 68 | Again, TLI is below the cutoff for a good model fit (.95) according to Hu & Bentler (1999), this should be stated and discussed more clearly! | We included a statement pointing out to this matter. |
| 69 | Discuss the TLI value > 1 for the ECS short in study 2. | We included a brief discussion about this matter as well. |
| 70 | Was ordinal alpha and omega calculated here? | We now added the ordinal alpha and omega values to the normal computations. |
| 71 | Explain why you consider 4 response options ordinal and 5 continuous (enough)? | Thank you for highlighting this point. We now added the ordinal alpha and omega values also for studies 2 and 3, in which five response options were used, to avoid confusion. |
| 72 | Summarize the descriptive statistics and psychometric properties in the text and report them in a table instead of the supplement. | We included the table in the manuscript and now summarize the information in the text. |
| 73 | Discuss the model fit of the configural invariance model more thoroughly (RMSEA and CFI are not good) and also report SRMR and TLI. | We also computed SRMR and TLI and discuss the fit of the configural model more critically. |
| 74 | Justify your choice of delta CFI as index for invariance (why not delta chi square or delta RMSEA)? | We chose delta CFI, since this is the criterion most often used for invariance testing. We revised our wording in the manuscript to more explicitly state this matter, referring to a source. |
| 75 | Be consistent in your use of the terms, eg residual or strict invariance, in the text and tables. Be consistent in how you report "rel. int. 05, ...", either in parenthesis or after a comma. | We now consistently use the term “strict invariance” instead of “residual invariance” and consistently report “rel. int.05, …” after a comma. |
| 76 | Justify why it seems more acceptable to you to modify the original response scale (4 options) than to keep it, given that internal consistency only "increased slightly". | It is true that the increase was not very large. However, the theoretical argument that five options (including a middle option) could be beneficial lead us to maintain the five categories. We included an explanation of this argument. |
| 77 | Does the ethics approval only pertain to study 3? Were study 1 and 2 also approved by the ethics committee? Please clarify. | We obtained explicit ethics approvals for both Study 2 and Study 3. We now inserted a statement clarifying that we obtained such an approval also for study 2 in the method section of study 2. We did not request a separate approval specifically for Study 1, but Study 1 is conceptualized parallel to Study 3 (both are online surveys with established personality questionnaires). |
| 78 | Explain why the preregistration was registered on april 8 and the data collection started in april 4 2024, but it says "preregistered before data collection" in the text. | Thank you for noticing this. The data collection indeed started on April 08, 2024, shortly after the registration was completed. April 04 was a mistake, which we now corrected in the manuscript. I have double checked the start date of the collection process, which I can find in the raw data frame retrieved from the online platform where the study was hosted: It was in the afternoon of april 08, 2024. The registration was uploaded earlier on that day and thus it was indeed submitted before data collection. |
| 79 | Discuss the large share of women in the sample (in all three studies). Consider conducting sensitivity analyses for all studies regarding participants' gender. | We considered to run sensitivity analyses for participants gender, but decided against reporting them due to the complexity of the analyses (especially the factor models, that were the focus of the manuscript) and the simultaneously low number of men included in our studies: For example, in study 1, 36 men participated, which does not allow us to specify and test our factor models separately for the genders and compare them accordingly. However, we now address the large share of women in our samples in the limitations section of the general discussion and point out to gender sensitivity analyses as an interesting potential future avenue. |
| 80 | Clarify whether all response options were presented to the participants with words or only the extremes (for all measures). | We revised the description of the response scales for all measures in all studies in order to avoid any confusions. |
| 81 | Add example items for all measures in all studies. | We added example items for all measures in all studies. |
| 82 | Again TLI below .95 is not excellent. | We revised the interpretation of the CFA results consistently throughout our manscript. |
| 83 | Report the AIC and compare the models accordingly (for all studies and model comparisons). | We added AIC values and discussed them. |
| 84 | In the preregistration it says that one item was reformulated and tested. I cant find anything on that analysis step in the manuscript. Please clarify. | Indeed, we reformulated one item (03) due to a potential problem associated with its content. However, the reformulation did not result in improved psychometric properties (internal consistency or item-total correlation) and therefore this item change was discarded for the final questionnaire (which contains the original item version). We had excluded this matter from the manuscript, since we deemed it not relevant for the reader. However, we understand that it is *lege artis* to report any expectation in full detail. Therefore, we inserted the whole issue (including its argumentation, analysis, results, and discussion again to increase transparency. |
| 85 | The potential influence of low alpha values (eg as low as .19) on the correlations should be discussed. | We included a discussion of the very low consistency of the Reading the Mind in the Eyes Test. It is a known problem of this measure and points out that the correlation to this test should be interpreted with caution. We included a statement clarifying this matter. |
| 86 | Better report and discuss confidence intervals for all correlations in all studies. | Thanks for this suggestion as well. We now added the confidence intervals to our results. We therefore extended the tables presenting the correlational results. In order to increase readability, we now present more tables (for the three studies and the two scales separately). |
| 87 | Overall, consider summarizing results and discussion in a combined "Results & Discussion" section (for all studies). | We combined the results and discussion sections in all three studies. |
| 88 | General Discussion: The dimensionality section of the discussion needs to be revised: The model fit of the 1F+CE model was not "close to the threshold for a good fit in studies 1 and 3" and the TLI was not excellent either in study 2. The model fit of the BF model was only excellent in study 2, but not in study 1 and 3 (TLI < .95). The findings should be discussed accordingly. | We revised the interpretation of CFA results according to your suggestions. |
| 89 | The model comparisons should be based on the AIC. The results should be reported transparently and discussed accordingly. | We computed and discussed AIC values (which though did not produce results leading to conclusions diverging from chi square). |
| 90 | In the convergent and discriminant validity section of the discussion, a concluding remark is missing regarding your evaluation of the scales' validity. | We added a concluding remark at the end of this section. |
| 91 | The longitudinal analyses section of the discussion might need to be revised depending on the revised findings, especially regarding the missing model fit indices. | We computed the other fit indices and revised our interpretation and discussion of the invariance section accordingly. |
| 92 | Emotional contagion is not a personality trait (line 631). It is an interpersonal process. What you are investigating is an individual's susceptibility to emotional contagion. | We agree and consequently renamed our core construct in the whole manuscript. |
| 93 | In line 632, "Hatfield et al." seems to be wrong. | Thanks for noting this, we deleted the phrase. |
| 94 | The limitations section of the discussion is very brief: Discuss the risk of alpha inflation due to multiple testing for all studies more thoroughly. Discuss the samples' homogeneity in all three studies more thoroughly (women, profession, etc). Discuss the different time points of the three studies with respect to the COVID-19 pandemic more thoroughly (before, during, and after the pandemic) and discuss the potential impact of the time point of study 2 (2016) being 6/8 years before the time points of studies 1 and 3. Discuss the lack of preregistrated analyses in study 1 and 2. | Thank you very much, we expanded the limitations section, now pointing out to the risk of alpha error inflation, which we though now also addressed by reporting FDR adjusted significance levels. We also added a more detailed discussion of the samples’ homogeneity and the potential impact of COVID-19 (and other crises) on the results, which surely may complicate comparisons between the studies, from which the earliest was conducted in 2016 and the latest in 2024. Finally, we also explicitly pointed out to the fact that studies 1 and 2 were not preregistered. |
| 95 | Include practical implications of your research in the discussion. | We added a discussion of implications and practical applications of the present research at the end of the discussion. |
| 96 | Language and Style: Some commas are missing. Sometimes blank spaces are missing between parentheses. APA level 2 headings are written in title case. Paragraphs should be indented consistently. Sentences should not start with numbers. | Thank you for your careful suggestions. We carefully searched for any mistakes as indicated and corrected them consistently throughout the manuscript.  With respect to the headings, we did not follow the APA guidelines, since the journal has specific guidelines, which state “Headings should be written in sentence case (capitalize only the first word of the heading, the first word of the subheading, and any proper nouns and genus names).” (see the “sample manuscript body (PDF)”, <https://journals.plos.org/plosone/s/submission-guidelines> ). |
