# Supplementary material for: Validation of the German Emotional Contagion Scale and development of a mimicry brief version
Source: PLoS One. 2025 Sep 9;20(9):e0331953. doi: 10.1371/journal.pone.0331953 (PMC12419621; doi:10.1371/journal.pone.0331953)
Supplement: Attachment — Submitted filename: Response_to_Reviewers_auresp_2.docx [file pone.0331953.s005.docx]

|  | **Editor‘s / Reviewer’s suggestions** | **Response** |
| --- | --- | --- |
|  | **Journal requirements** |  |
| 01 | If the reviewer comments include a recommendation to cite specific previously published works, please review and evaluate these publications to determine whether they are relevant and should be cited. There is no requirement to cite these works unless the editor has indicated otherwise. | Thank you for this clarification. The previous reviewer comments recommended us to provide the reader with a more comprehensive literature overview, but did not request specific citations from us. The sources we added in the first revision were deemed relevant and chosen freely by us. |
| 02 | Please review your reference list to ensure that it is complete and correct. If you have cited papers that have been retracted, please include the rationale for doing so in the manuscript text, or remove these references and replace them with relevant current references. Any changes to the reference list should be mentioned in the rebuttal letter that accompanies your revised manuscript. If you need to cite a retracted article, indicate the article’s retracted status in the References list and also include a citation and full reference for the retraction notice. | We have reviewed our reference list. We did not find any retracted articles or other mistakes. We included a detailed list of any changes made to the reference list in this rebuttal letter (see below). This list of reference changes includes as well the changes made in the previous revision, as changes made in the current revision. |
|  | **Reviewer #1:** |  |
|  | The authors have addressed all the reviews and comments, and I believe that the paper, in its current form, is of sufficient quality to be published in the journal PLOS One. | We thank you very much for your time and we are happy that you deem our manuscript suitable for publication. |
|  | **Reviewer #2** |  |
|  | Overall. Thank you for the careful and extensive response to reviewer comments. As with the initial submission, I find your research very interesting and quite likely valuable theoretically and practically. I appreciate the theoretical framework and justification for the three research studies. Overall, the research in my opinion is measurement development and validation with strengths and weaknesses. But, the missing analyses do not render your findings and interpretations indefensible. I do have lingering comments and questions regarding (a) formatting, and (b) unsubstantiated interpretations. | Thank you again very much for reading and reviewing our manuscript and for the appreciation you expressed. |
| 03 | Title. Comment. The Full Title is excessive, the Short Title is preferred and sufficiently accurate. | We changed the title as requested. |
| 04 | Keywords. Comment. Because your measures focus on 'mimicry', I would consider including 'mimicry' as a keyword. Rather than validity, reliability, and factor structure, consider dropping 'factor structure', and/or replace all terms with 'psychometrics'. | We added “mimicry” as a keyword and generally revised the keywords in light of the shortened title. |
| 05 | Abstract. The abstract is sufficiently comprehensive. Reference to three studies is made, but only Study 2 is mentioned specifically. The acronym 'sECS' is used, but not defined.  1. Recommendation. Do not mention any of the three studies, specifically. Consider not mentioning specific findings, e.g., "The correlation pattern . . . and longitudinal invariance." Perhaps it suffices to state that three studies using psychometric modeling and construct validation procedures concluded emotional contagion is essentially unidimensional, with secondary dimensions. Using these findings . . . a short 'mimicry' scale was developed and validated. And so on . . .. | Thank you for highlighting this point. We omitted the abbreviation “sECS” from the abstract. We also avoided to merely refer to study 2 specifically. However, we maintained a description of the most important specific findings, which also include exceptional analyses beyond the typical psychometric validation procedures, which may be unexpected by and of special interest for the reader (e.g., longitudinal invariance and stability). |
| 06 | Overall. The theoretical framework is clearly provided. Essential constructs and variables are identified along with the purpose(s) of the current research. 1. Comment. You start with the constructs 'emotional contagion' (and components including 'mimicry'), and 'empathy', and 'susceptibility to emotional contagion'. It is the susceptibility to emotional contagion as a personality trait that underlies the argument that measurement of 'mimicry' is meaningful. | Thank you for the appreciation you expressed. In the previous revision, we had restructured the introduction to address another reviewer’s comments recommending a more thorough introduction into the background of the core construct (susceptibility to emotional contagion). We now added a sentence at the beginning of the introduction, clearly stating that the present research indeed focuses on the susceptibility to emotional contagion, but emotional contagion is at first defined for the purpose of a thorough introduction into the topic. |
| 07 | 2. The transition to a measure of 'mimicry' as though it is a measure of 'susceptibility of emotional contagion' is critical. This is made most clear in two places. First, you state, "Specifically, SEC can be defined as "the tendency to automatically mimic and synchronize expressions, vocalizations, postures, and movements with those of another person's and consequently, to converge emotionally". (lines 121-123) Second, the section 'Content examination of the ECS items' explains, "The ECS contains items tapping the basic process of mimicry." (line 207). | Thank you for emphasizing this point as well. We agree that this is a crucial matter, since the relation between mimicry and SEC is complex. Both are surely two different terms, but the tendency to express mimicry can be regarded a subconstruct of SEC. Most importantly, mimicry refers to the most important and fundamental and overt part of SEC, rendering it an especially interesting potentially brief measurement approach to the total SEC construct.  In order to consequently implement the conceptualization of SEC as a basal and “primitive” trait, we decided to solely focus on items explicitly tapping this process in the short scale. We added a sentence in the section “Susceptibility to emotional contagion” as well as sentences in the section “The present studies” to clarify this matter. To illustrate our argumentation, we also inserted a short comparison to Raven’s matrices, which are conceptualized as measuring basic processes underlying the *g* factor of intelligence. |
| 08 | 3. Suggestion. Make more clear why 'mimicry' measurement is relevant, if not identical to susceptibility of emotional contagion. Perhaps the statement above (comment 2) intends this, but the transition to 'mimicry' as the focal measure is lost when you persist with the SEC scaling discussion in 'The present studies' section. Regarding your selection of mimicry items, you state, ". . . they capture mimicry, the first state of SEC, which can be considered to reflect SEC in the most basic way." (lines 271-272). You do further explain the significance of mimicry, but it may be helpful to do this in the theoretical framework when justifying development of a 'mimicry' measure. | As explained above, we added a more thorough explanation of why the mimicry items appear to be of special interest for the measurement of SEC. We do not conceptualize mimicry as an independent phenomenon of SEC – both are, as described, strongly overlapping. Surely, mimicry only refers a subprocess of SEC, yet to the most important and the most fundamental and overt subprocess and is thus of special interest to investigate. |
| 09 | The Series of Three Studies Collectively. Methods. Overall. Your methods are generally complete with ample description of the models and modeling procedures. 1. Comment. Study 1 uses the four-point Likert scale, Studies 2 and 3 use a five-point Likert scale as described, and a sum of item responses is the total scale score for both the ECS and Mimicry measures. This modified item response scaling is critical for reasons you mention. One analysis you did not do, which would be interesting, is the study of the responses using the so called 'neutral' response. As suggested in prior comments, an item response modeling of the item responses would be interesting and you do mention this in your Discussion. You use this item response format as one possible explanation for the reliability and correlations obtained in Studies 2 and 3, relative to the relatively low values in Study 1. | Thank you for your comment. We have now conducted IRT analyses, i.e., we specified a partial credit model and item-wise plotted the category characteristic curves (CCCs). The results indicated that the CCC of the middle response category was in each case between the CCCs of the two adjoint response categories, suggesting that the neutral response is used validly by the participants (a source is cited for this interpretation). Since the paper already contains many findings, analyses, and tables and since the response format is not the focus of the paper, we decided to report the detailed findings (plots) in the supplementary material and to only briefly mention and discuss them in the main text. |
| 10 | 2. Comment. For each study you have a section 'Internal consistency'. Consider 'Reliability' rather than 'internal consistency', even though you do use coefficient alpha. The focus of the section is on 'reliability' and the specific index. Perhaps expand the discussion to focus on item and total score reliability. | We renamed the sections from “Internal consistency” to “Reliability”. We also added a brief discussion of the item statistics, but decided to keep this part short in light of the length of the paper and analyses. |
| 11 | Results. Overall. Results from analyses per study are well described and interpretation are accurate. I simply wish you had completed more item level analyses given what you think are critical implications of the item response format (4, 5 point Likert scale). | Thank you for the appreciation you expressed. As explained above, we now added item-level analyses (IRT), but – in light of the length of the manuscript and the extent of the already reported analyses and findings – we tried to keep this addition concise. |
| 12 | Discussion. Overall. There are many findings to be discussed and you were quite thorough. One topic I found questionable pertained to the sample/populations you studied. Irrespective of whether you are sampling students or nurses, the actual psychometrics for ECS and mimicry should reasonably be invariant. I cannot imagine why factor structure, correlations, etc. would vary across these populations unless the measures are unreliable, which they actually are, i.e., unreliable. The low reliability is your best explanation for the variance across these populations. You did obtain acceptable convergent and discriminant validity evidence, which is somewhat unexpected given the low reliability, particularly in Study 1 with the Mimicry measure. | Thanks again for highlighting this point. We added a statement in the limitations section of the general discussion, pointing out to the variance between the findings between the three studies / samples, which can be explained by the low reliability of the measures. |
| 13 | Writing. Overall. The manuscript is very carefully written. 1. Major Recommendation. My primary concern pertains to the formatting within the presentation of the three studies. The bold headings and subheadings make it very difficult to appreciate the nested content organization. I suggest uses a heading and subheading formatting that clearly differentiates subheads/content. | We revised our formatting to make the differences between adjoining levels of headings more distinct (bold 18pt, bold italics 16pt, bold 14 pt, italics 12 pt) to avoid any confusions. |
| 14 | 2. Comment. Often technical terms are inconsistent with conventional nomenclature. Here are few examples. (a). line 175 'obstructing the fit' (maybe terms such as 'attenuating', 'diminishing' the fit) are more conventional. (b) line 220 'love should be counted as a basic emotion' (love should be considered . . .) (c) line 234 'version a psychological meaning' (version a construct validation) | We reviewed our manuscript and corrected any formulations that seemed incompatible with conventional nomenclature, including the examples you described. |
| 15 | 3. Comment. There are occasional spelling errors, for example, (a) page 12 line 266 spelling error 'suceptiblity'. | We proofread our manuscript and corrected any spelling mistakes. |
|  |  |  |
|  | **Additional Changes** |  |
|  | Changes to the reference list (made in the previous revision) | We added the following references:  Becker H. Some forms of sympathy: a phenomenological analysis.DP - Apr 1931. The Journal of Abnormal and Social Psychology. 1931;26(1):58-68.  Sullins ES. Emotional contagion revisited: Effects of social comparison and expressive style on mood convergence.DP - Apr 1991. Personality and Social Psychology Bulletin. 1991;17(2):166-74.  Hess U, Fischer A. Emotional mimicry: Why and when we mimic emotions. Social and personality psychology compass. 2014;8(2):45-57.  Herrando C, Constantinides E. Emotional contagion: A brief overview and future directions. Frontiers in Psychology Vol 12, 2021, ArtID 712606. 2021;12.  Harada T, Hayashi A, Sadato N, Iidaka T. Neural correlates of emotional contagion induced by happy and sad expressions. Journal of Psychophysiology. 2016;30(3):114-23.  Lin D, Zhu T, Wang Y. Emotion contagion and physiological synchrony: The more intimate relationships, the more contagion of positive emotions. Physiology & Behavior. 2024;275:114434.  Mayo O, Horesh D, Korisky A, Milstein N, Zadok E, Tomashin A, et al. I feel you: Prepandemic physiological synchrony and emotional contagion during COVID-19. Emotion. 2023;23(3):753-63.  Homan MD, Schumacher G, Bakker BN. Facing emotional politicians: Do emotional displays of politicians evoke mimicry and emotional contagion? Emotion. 2023;23(6):1702-13.  Hall JA, Schwartz R. Empathy, an important but problematic concept. The Journal of Social Psychology. 2022:1-6.  Gerdes KE, Segal EA, Lietz CA. Conceptualising and measuring empathy. British Journal of Social Work. 2010;40(7):2326-43.  Cuff BM, Brown SJ, Taylor L, Howat DJ. Empathy: A review of the concept. Emotion review. 2016;8(2):144-53.  Altmann T, Roth M. The evolution of empathy: From single components to process models. Handbook of psychology of emotions. 2013;2:171-87.  Marx AKG, Frenzel AC, Fiedler D, Reck C. Susceptibility to positive versus negative emotional contagion: First evidence on their distinction using a balanced self-report measure. PLOS ONE. 2024;19(5):e0302890.  Kevrekidis P, Skapinakis P, Damigos D, Mavreas V. Adaptation of the Emotional Contagion Scale (ECS) and gender differences within the Greek cultural context. Annals of General Psychiatry Vol 7, 2008, ArtID 14. 2008;7.  Ekman P. An argument for basic emotions. Cognition and Emotion. 1992;6(3-4):169-200.  Barrett LF. Are Emotions Natural Kinds? Perspectives on Psychological Science. 2006;1(1):28-58.  Colombetti G. From affect programs to dynamical discrete emotions. Philosophical Psychology. 2009;22(4):407-25.  Marsh HW, Hau K-T, Wen Z. In search of golden rules: Comment on hypothesis-testing approaches to setting cutoff values for fit indexes and dangers in overgeneralizing Hu and Bentler's (1999) findings. Structural equation modeling. 2004;11(3):320-41.  Burnham KP, Anderson DR. Multimodel Inference:Understanding AIC and BIC in Model Selection. Sociological Methods & Research. 2004;33(2):261-304.  Bearden WO, Sharma S, Teel JE. Sample Size Effects on Chi Square and Other Statistics Used in Evaluating Causal Models. Journal of Marketing Research. 1982;19(4):425.  Lienert GA, Raatz U. Testaufbau und Testanalyse: Beltz Verlagsgruppe; 1998.  Kusmaryono I, Wijayanti D, Maharani HR. Number of response options, reliability, validity, and potential bias in the use of the likert scale education and social science research: A literature review. International Journal of Educational Methodology. 2022;8(4):625-37.  Benjamini Y, Hochberg Y. Controlling the False Discovery Rate: A Practical and Powerful Approach to Multiple Testing. Journal of the Royal Statistical Society Series B (Methodological). 1995;57(1):289-300.  Brunero S, Lamont S, Coates M. A review of empathy education in nursing. Nursing Inquiry. 2010;17(1):65-74.  Bentler PM, Bonett DG. Significance tests and goodness of fit in the analysis of covariance structures. Psychological Bulletin. 1980;88(3):588-606.  Reniers RLEP, Corcoran R, Drake R, Shryane NM, Völlm BA. The QCAE: A Questionnaire of Cognitive and Affective Empathy. Journal of Personality Assessment. 2011;93(1):84-95.  Gomez R, Brown T, Watson S, Stavropoulos V. Confirmatory factor analysis and exploratory structural equation modeling of the factor structure of the Questionnaire of Cognitive and Affective Empathy (QCAE). PLOS ONE. 2022;17(2):e0261914.  Wheaton MG, Prikhidko A, Messner GR. Is Fear of COVID-19 Contagious? The Effects of Emotion Contagion and Social Media Use on Anxiety in Response to the Coronavirus Pandemic. Frontiers in Psychology. 2021;Volume 11 - 2020.  Guhn A, Merkel L, Hübner L, Dziobek I, Sterzer P, Köhler S. Understanding versus feeling the emotions of others: How persistent and recurrent depression affect empathy. Journal of Psychiatric Research. 2020;130:120-7.  Altmann T, Roth M. The risk of empathy: longitudinal associations between empathy and burnout. Psychol Health. 2021;36(12):1441-60.  Joiner TE. Contagious depression: Existence, specificity to depressed symptoms, and the role of reassurance seeking. Journal of Personality and Social Psychology. 1994;67:287-96.  Asendorpf JB. Persönlichkeitspsychologie für Bachelor. 5th 2024 ed. Berlin, Heidelberg: Springer Berlin Heidelberg; 2024.  We did not delete any references. |
|  | Changes to the reference list (made in the current revision) | References we added:  Kankaraš, M., & Capecchi, S. (2025). Neither agree nor disagree: use and misuse of the neutral response category in Likert-type scales. *METRON*, *83*(1), 111-140. https://doi.org/10.1007/s40300-024-00276-5  Mackintosh, N. J., & Bennett, E. S. (2005). What do Raven's Matrices measure? An analysis in terms of sex differences. *Intelligence*, *33*(6), 663-674. https://doi.org/https://doi.org/10.1016/j.intell.2005.03.004  We did not delete any references. |
